# *In vitro* antimicrobial activity and resistance mechanisms of cefiderocol against clinical carbapenem-resistant gram-negative bacteria

**DOI:** 10.3389/fmicb.2025.1670179

**Published:** 2025-10-03

**Authors:** Ruyu Yan, Jun Ji, Liya Wang, Yuerong Zou, Han Shen, Jinhua Yuan, Xiaoli Cao

**Affiliations:** ^1^Department of Laboratory Medicine, Nanjing Drum Tower Hospital Clinical College of Nanjing University of Chinese Medicine, Nanjing, Jiangsu, China; ^2^Nanjing Municipal Centre for Disease Control and Prevention, Nanjing Municipal Key Laboratory of Public Health Testing, Nanjing, Jiangsu, China; ^3^Jiangsu Key Laboratory of Laboratory Medicine, Department of Immunology, School of Medicine, School of Chemistry and Chemical Engineering, Jiangsu University, Zhenjiang, China

**Keywords:** cefiderocol, carbapenem-resistant gram-negative bacteria, antimicrobial susceptibility, resistance mechanisms, efflux pump inhibitor, mutation

## Abstract

**Background:**

The rise of carbapenem-resistant gram-negative bacteria (CRGNB) necessitates new therapeutic options such as cefiderocol.

**Objective:**

To evaluate the *in vitro* efficacy of cefiderocol against clinical CRGNB and investigate associated resistance mechanisms.

**Methods:**

A total of 370 CRGNB isolates were analyzed. Minimum inhibitory concentration (MIC) values were determined, and whole genome sequencing, efflux pump inhibition assays, and RT-qPCR were conducted to assess resistance-related mutations, gene loss, and expression changes.

**Results:**

Cefiderocol demonstrated potent *in vitro* activity, with high susceptibility rates in *C. freundii* (100%), *K. pneumoniae* (93.3%), and *E. hormaechei* (92.2%), and notable activity against *P. aeruginosa* (80.0%) and *Escherichia coli* (76.8%). Efflux pump inhibition by Carbonyl Cyanide m-Chlorophenyl Hydrazone (CCCP) significantly reduced MICs in resistant strains. Key resistance mechanisms included *β*-lactamase gene variants (*bla*_OXA-66_, *bla*_OXA-23_, *bla*_SHV-12_), mutations in *envZ*, *cirA*, *nuoC*, *ampC*, and loss or altered expression of iron transporter genes (*piuA*, *pirA*, *fepA*).

**Conclusion:**

Cefiderocol is highly effective against CRGNB; however, resistance may arise through diverse mechanisms, including efflux pump activity. Continued surveillance of emerging resistance is essential to guide its optimal clinical use.

## 1 Introduction

Carbapenem-resistant Gram-negative bacteria (CRGNB) have emerged as a significant global public health threat, posing substantial challenges in healthcare settings (Daoud and Dropa, 2023). These pathogens, including carbapenem-resistant *Enterobacterales* (CRE), carbapenem-resistant *Pseudomonas aeruginosa* (CRPA), and carbapenem-resistant *Acinetobacter baumannii* (CRAB), are associated with high morbidity and mortality due to limited therapeutic options and their ability to rapidly acquire and disseminate resistance genes, such as those encoding *β*-lactamases (Bassetti et al., 2021).

The global prevalence of CRGNB continues to rise, with notable regional variations (Jean et al., 2022). In Europe and North America, the incidence of CRE and CRAB has significantly increased, particularly in healthcare-associated infections (Müller et al., 2023; Duffy et al., 2023). In Asia, especially in countries like China and India, the spread of CRGNB has been exacerbated by high antibiotic consumption and inadequate infection control measures (Yin et al., 2023; Vijay et al., 2021). The Middle East and Latin America are also experiencing a growing burden of CRGNB infections, often linked to nosocomial outbreaks (Sha et al., 2017; Garcia-Betancur et al., 2021). These bacteria challenge healthcare systems worldwide, leading to prolonged hospital stays, increased healthcare costs, and limited therapeutic options (Li et al., 2024).

**Figure 1:**
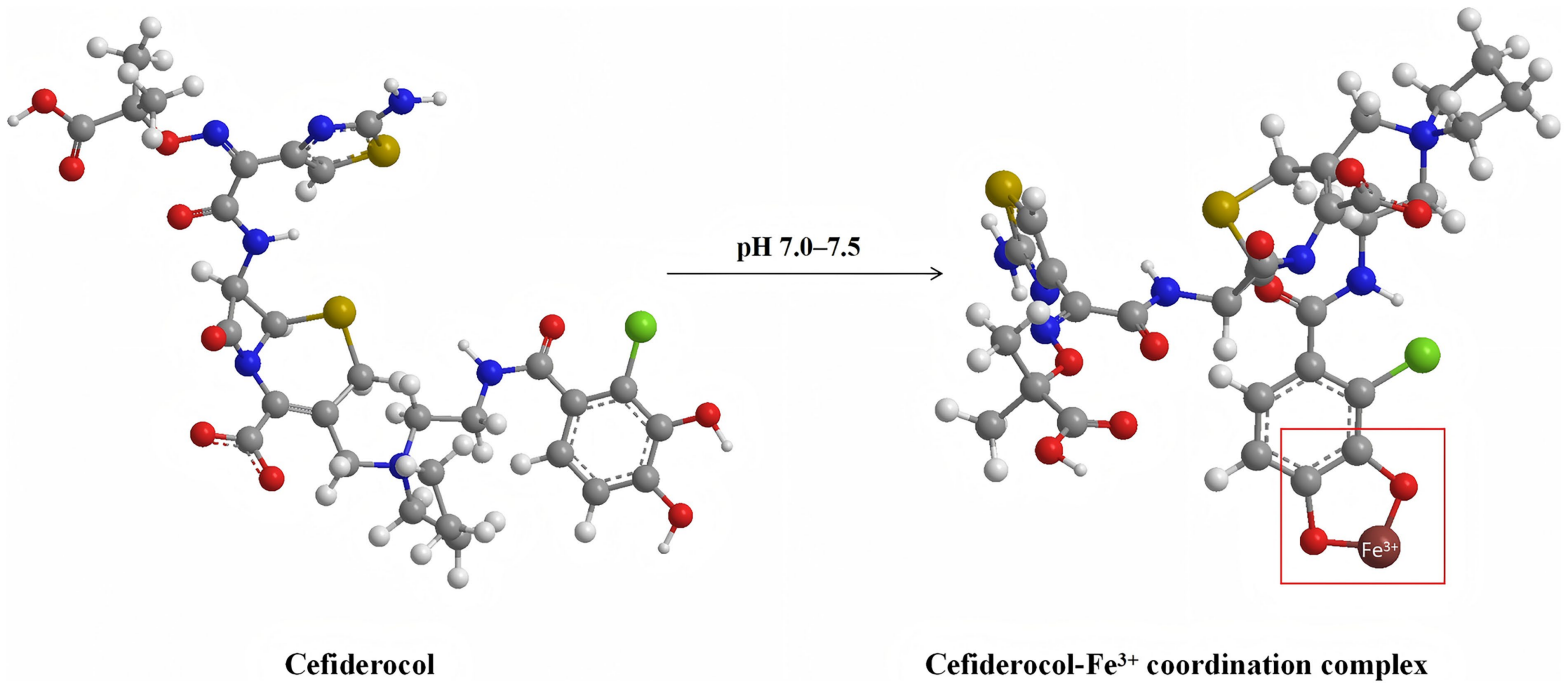
Molecular structures of cefiderocol (left) and its ferric iron (Fe^3+^) coordination complex (right) at physiological pH (7.0–7.5). This schematic depicts the chelation of cefiderocol with ferric iron, a critical step enabling its active uptake through bacterial iron transport systems. Atom colors are as follows: carbon (gray), oxygen (red), nitrogen (blue), sulfur (yellow), and iron (brown). The arrow indicates the pH-dependent formation of the cefiderocol–Fe^3+^ complex. The molecular model is the 3D structure described by MolView (https://molview.org).

In response to the rising antimicrobial resistance, the development and evaluation of novel therapeutic agents have become urgent priorities. Cefiderocol, a novel siderophore cephalosporin antibiotic, employs a unique mechanism of action (as illustrated in Figure 1). By exploiting the bacterial iron transport system, cefiderocol can penetrate the outer membrane of Gram-negative bacteria (Ong'uti et al., 2022). This “Trojan horse” strategy allows cefiderocol to bypass common resistance mechanisms, such as alterations in porin channel and efflux pumps, which often limit the efficacy of other *β*-lactam antibiotics (Karakonstantis et al., 2022). Cefiderocol has been reported to be particularly effective against CRGNB, demonstrating stability against a wide array of *β*-lactamases, including metallo-β-lactamases and serine carbapenemases, thus enhancing its potential in treating severe infections caused by resistant pathogens (Wang et al., 2022; Sollima et al., 2024).

Up to date, resistance mechanisms to cefiderocol include enzyme-mediated hydrolysis (e.g., metallo-*β*-lactamases and certain serine β-lactamases), mutations in porins leading to reduced outer membrane permeability, efflux pump overexpression, and modifications of target sites. Additionally, novel resistance determinants and adaptive mutations under antibiotic pressure have been reported. Notably, the amplification of specific β-lactamase genes, such as *bla*_SHV-12_, correlates with elevated resistance in *Klebsiella pneumoniae* (Moon and Huang, 2023). Altered membrane permeability exacerbates resistance through various mechanisms: mutations in iron transport-associated loci (*envZ*, *tonB*, *cirA*) reduce drug influx, while structural changes in the *piuC* gene (which encodes an outer membrane siderophore receptor) and regulatory mutations upstream of its operon impair cefiderophore uptake (Kriz et al., 2024). Porin mutations can increase cefiderocol Minimum inhibitory concentration (MIC) in isogenic *K. pneumoniae* (OmpK35, OmpK36) and *P. aeruginosa* (OprD) mutants (Ito et al., 2018). Enhanced efflux pump activity can expel cefiderocol, with target mutations in multidrug efflux regulators (such as *baeS*, *czcS*, *nalC*), antibiotic inactivation enzymes (*ampR*, *dacB*), and penicillin-binding protein genes (*mrcB*) playing key roles (Kriz et al., 2024).

Although cefiderocol has shown potent activity against CRGNB in previous studies (Yousefi et al., 2024; Piccica et al., 2023; de la Fuente et al., 2023), most investigations have been conducted outside of China, where resistance mechanisms and epidemiological patterns may differ. Regional data are therefore essential to assess whether these findings are generalizable to local clinical settings. In particular, little is known about the contribution of efflux pumps, resistance gene expression, and genomic variations to cefiderocol resistance among Chinese isolates.

To address this gap, the present study evaluated the *in vitro* antimicrobial activity of cefiderocol against a large collection of clinical CRGNB isolates, including CRE, CRPA, and CRAB. In addition, we investigated the role of efflux pumps through inhibition assays, quantified the expression of resistance-associated genes using RT-qPCR, and applied whole-genome sequencing (WGS) to identify gene mutations and loss events linked to cefiderocol resistance.

The objective of this study was to provide localized evidence on the efficacy and resistance mechanisms of cefiderocol in China, thereby contributing to both clinical decision-making and the global understanding of its role in managing CRGNB infections.

## 2 Materials and methods

### 2.1 Strain collection

A total of 370 CRGNB isolates were tested, including 58 *K. pneumoniae*, 99 *Escherichia coli*, 7 *K. oxytoca*, 51 *E. hormaechei* and 20 *C. freundii*, 82 *A. baumannii*, 53 *P. aeruginosa.* Due to the clonal dissemination of Carbapenem-resistant *K. pneumoniae* (CRKP) in our hospital, the major *K. pneumoniae* strains were collected from 15 hospitals in Nanjing city. The remaining strains were isolated from Nanjing Drum tower hospital. Among them, 99 *E. coli*, 7 *K. oxytoca*, 51 *E. hormaechei*, 20 *C. freundii*, and 53 *P. aeruginosa* were isolated between 2013 and 2021, and 82 *A. baumannii* strains were isolated between 2019 and 2022. All bacterial strains were obtained from various clinical specimens such as blood (*n* = 157), sputum (*n* = 75), urine (*n* = 63), secretion (*n* = 21), abdominal dropsy (*n* = 21), bile (*n* = 12), specimens with missing clinical source information (*n* = 8), catheter (*n* = 4), pleural effusion (*n* = 3), cerebrospinal fluid (*n* = 2), pus (*n* = 2), rectal swab (*n* = 1), and incision secretion (*n* = 1). The detailed distribution of isolation sources for each bacterial species is summarized in Supplementary Table S1.

### 2.2 Preparation of iron-depleted cation-adjusted Mueller–Hinton broth (ID-CAMHB)

Cefiderocol susceptibility testing was performed using iron-depleted cation-adjusted Mueller–Hinton broth (ID-CAMHB), prepared in accordance with Clinical and Laboratory Standards Institute (CLSI) 2024 guidelines (Clinical and Laboratory Standards Institute (CLSI), 2023). Briefly, 21.0 g of Mueller–Hinton (MH) medium was dissolved in 1 L of purified water, sterilized by autoclaving at 121 °C for 15 min, and cooled to room temperature. Chelex^®^ 100 resin (100 g) was added, and the suspension was stirred with a magnetic stirrer for 6 h at room temperature. Following centrifugation, the supernatant was collected, and iron concentration was determined by inductively coupled plasma optical emission spectrometry (ICP-OES). When the iron concentration was confirmed to be < 0.03 μg/mL, the pH was adjusted to 7.2–7.4, and the medium was sequentially supplemented with ZnSO₄·7H₂O (final concentration 0.5–1.0 μg/mL), CaCl₂ (20.0–25.0 μg/mL), and MgCl₂·6H₂O (10.0–12.5 μg/mL). The final medium was sterilized by filtration through a 0.22 μm membrane, designated as ID-CAMHB, and stored at 4 °C until use.

### 2.3 MIC determination

Antimicrobial susceptibility testing was conducted using microbroth dilution for the following antibiotics: imipenem, ceftazidime, ceftriaxone, cefepime, amikacin, ciprofloxacin, levofloxacin, tobramycin, and gentamicin. Cefiderocol susceptibility was determined using iron-depleted cation-adjusted Mueller-Hinton broth, with a cefiderocol concentration range of 0.03125–32 μg/mL. *E. coli* ATCC25922 was used as a quality control. Results were interpreted according to CLSI2024 guideline (Clinical and Laboratory Standards Institute (CLSI), 2023).

### 2.4 Analysis of gene acquisition, mutations, and loss

WGS was performed on all 370 CRGNB isolates to investigate the genetic basis of cefiderocol resistance. Genomic DNA was extracted using standard protocols and sequenced using an Illumina platform. High-quality reads were assembled *de novo*, and annotation was conducted using Prokka. Comprehensive bioinformatics analyses were employed to identify gene acquisition events, point mutations, and gene loss associated with cefiderocol resistance. Specifically, resistance-related genes, including *β*-lactamases and iron transporter-associated genes, were examined. Comparative genomic analyses between cefiderocol-resistant and -susceptible isolates were conducted to detect significant mutations and gene deletions with *A. baumannii* ATCC 19606 (Accession Number: CP058289.1), *E. coli* K-12 MG1655 (Accession Number: NC_000913.3), *K. pneumoniae* ATCC 13883 (Accession Number: KN046818.1), and *P. aeruginosa* PAO1 (Accession Number: AE004091.2) being as reference genomes. Identified genetic alterations were further analyzed to elucidate their potential role in the emergence of cefiderocol resistance. The corresponding accession numbers for all sequenced isolates, including both cefiderocol-resistant and cefiderocol-susceptible strains, are now provided in Supplementary Table S2. WGS data of the cefiderocol-resistant isolates have been deposited in the NCBI GenBank database. The accession numbers are as follows: *P. aeruginosa*: 4087 (JBBXJG000000000), 19,686 (JBBXHS000000000), 12,218 (JBBXIN000000000), 17,774 (JBBXHW000000000), 3,077 (JBBXJJ000000000), 2,745 (JBBXJK000000000); *A. baumannii*: 10521 (JAVIKM000000000), 11,253 (JAVIKS000000000), 12,076 (JAVILL000000000), 12,150 (JAVILM000000000), 12,439 (JAVILT000000000), 14,118 (JAVIMK000000000), 14,179 (JAVIML000000000), 14,184 (JAVIMM000000000); *E. coli*: 3034 (JANWRX000000000), 14,449 (JANWPI000000000), 12,010 (JANWQC000000000), 14,109 (JANWPL000000000), 14,334 (JANWPJ000000000), 15,503 (JANWOY000000000), 16,769 (JANWOK000000000); *E. hormaechei*: 1707 (JANWOH000000000), 14,787 (JANWMY000000000); *K. pneumoniae*: njsetyy13 (VEON00000000), njxkyy19 (RZKE00000000).

### 2.5 Inhibition of efflux pump activity by carbonyl cyanide m-chlorophenyl hydrazone (CCCP) and reserpine

The role of efflux pumps in CRGNB resistance was evaluated using the CCCP and Reserpine inhibition tests. MIC changes were assessed in the presence and absence of CCCP (MedChemExpress, China) and Reserpine (MedChemExpress, China) at a final concentration of 50 μg/mL. Each isolate was inoculated into ID-CAMHB containing serial dilutions of cefiderocol. Overexpression of the efflux pump was considered as a positive phenotype when cefiderocol MIC was reduced by at least fourfold in the presence of CCCP and reserpine.

### 2.6 Gene expression was analyzed by RT-qPCR

Total RNA was extracted from bacteria using the RNAEX reagent (Accurate Biotechnology, Hunan, China) and the SteadyPure Universal RNA Extraction Kit (Accurate Biotechnology) according to the manufacturer’s instructions. The extracted RNA was reversely transcribed into cDNA using the Evo M-MLV RT Premix for qPCR (Accurate Biotechnology, Hunan, China). RT-qPCR was performed using a qPCR kit (Accurate Biotechnology, China). Relative RNA expression levels were analyzed using the 2^−∆∆Ct^ method. All RT-qPCR analyses were conducted in triplicate biological replicates. The housekeeping gene used for *P. aeruginosa* was *rpoD*, while *rpoB* served as the housekeeping gene for both *A. baumannii* and *K. pneumoniae.* Primer sets are listed in Supplementary Table S3.

### 2.7 Construction of a phylogenetic tree

Assembled contigs in FASTA format from each sequencing run were submitted to the Centre for Genomic Epidemiology (CGE)^1^ (Kaas et al., 2014). Phylogenetic relationships were inferred using the web-based tool CSI Phylogeny 1.4 with stringent SNP filtering: minimum SNP quality of 30, mapping quality of 25, sequencing depth ≥10×, relative depth ≥10%, and Z-score ≥1.96. Heterozygous SNPs were optionally excluded, and a minimum distance of 10 bp between pruned SNPs was applied to reduce linkage bias. The tree was visualized using iTOL (Letunic and Bork, 2019).

### 2.8 Statistical analysis

Statistical analyses were performed using Microsoft Excel (Version 19.0, Microsoft Corporation, Redmond, WA, United States) and SPSS (Version 20.0). Descriptive statistics, including MIC_50_, MIC_90_, and MIC ranges, were calculated for all variables. Comparisons between groups with and without *β*-lactamase genes were performed using the non-parametric Mann–Whitney U test. Classification of cefiderocol-resistant and susceptible strains was analyzed using the Chi-squared test (with continuity correction) and Fisher’s exact test, while RT-qPCR data were evaluated using independent *t*-tests. Statistical significance was defined as *p* < 0.05. In figures, significance levels were indicated as follows: * p < 0.05, ** p < 0.01, *** p < 0.001, with non-significant differences marked as “ns.”

## 3 Result

### 3.1 Distribution and cumulative MIC of cefiderocol and other clinically used antibiotics against CRGNB isolates

**Figure 2:**
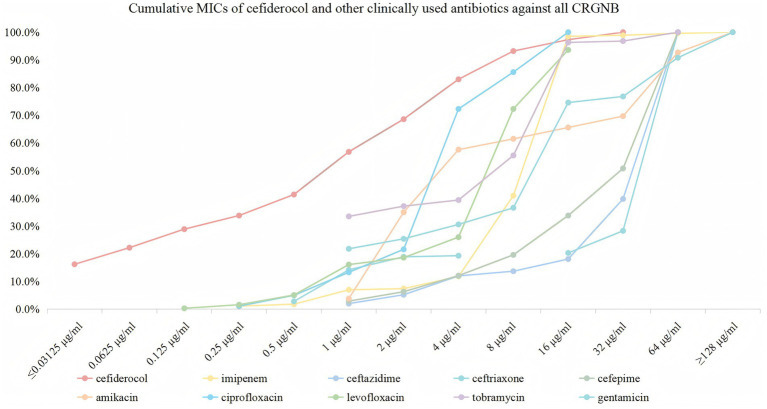
Cumulative MICs of cefiderocol and other clinically used antibiotics against all CRGNB. The figure illustrates the cumulative susceptibility of cefiderocol (red) and comparator antibiotics (imipenem, ceftazidime, ceftriaxone, cefepime, amikacin, ciprofloxacin, levofloxacin, tobramycin, and gentamicin) against CRGNB isolates. The x-axis represents MIC values (μg/ml) on a logarithmic scale (≤0.03125 to ≥128), and the y-axis shows the cumulative percentage of isolates inhibited at each concentration.

Cefiderocol exhibited high antibacterial activity against the 370 CRGNB isolates. Notably, 16.2% of the isolates were susceptible to cefiderocol, showing a very low MIC of ≤0.03125 μg/mL, and 68.6% of isolates were inhibited at 2 μg/mL. At 4 μg/mL, the inhibition rate increased to 83.0%, with 100.0% inhibition achieved at 32 μg/mL. In comparison, imipenem exhibited lower initial activity, inhibiting only 1.1% of isolates at 0.25 μg/mL. Significant inhibition was observed starting at 16 μg/mL, with 98.5% of isolates inhibited, and 100.0% inhibition at ≥128 μg/mL. Other *β*-lactams, such as ceftazidime, ceftriaxone, and cefepime, showed variable effectiveness, with notable inhibition only at higher concentrations. Aminoglycosides and fluoroquinolones exhibited better activity, but their efficacy was generally lower than that of cefiderocol. The detailed susceptible data, including resistance percentages, are provided in Table 1 and Figure 2.

**Table 1:** Antimicrobial activities of cefiderocol and comparator agents against all carbapenem-resistant gram-negative bacteria isolates.

Species (*n*)	Drug	MIC (ug/ml)			Susceptible	% of isolate	Resistant
		50%	90%	Range		Intermediate	
*Escherichia coli*	Cefiderocol	1	8	0.03125–32	76.8	16.2	7.1
	Imipenem	16	16	1–16	7.2	0	92.8
	Ceftazidime	64	64	1–64	2.4	0	97.6
	Ceftriaxone	64	64	1–64	1.2	0	98.8
	Cefepime	64	64	1–64	2.4	4.9	92.7
	Amikacin	2	64	2–64	70.2	3.6	26.2
	Ciprofloxacin	4	4	1–8	3.7	0	96.3
	Levofloxacin	8	8	0.5–8	3.6	3.6	92.9
	Tobramycin	8	16	1–16	6.1	9.8	84.1
	Gentamicin	16	16	1–16	35.4	3.7	61
*Enterobacter hormaechei*	Cefiderocol	0.125	4	0.03125–32	92.2	3.9	3.9
	Ceftazidime	64	64	32–64	0	0	100
	Ceftriaxone	64	64	32–64	0	0	100
	Cefepime	64	64	4–64	0	20.7	79.3
	Amikacin	16	64	2–64	36	4	60
	Levofloxacin	8	64	0.5–64	2	10.2	87.8
	Tobramycin	8	16	1–16	26.9	7.7	65.4
	Gentamicin	16	16	1–16	26.9	7.7	65.4
*Klebsiella pneumoniae*	Cefiderocol	0.5	4	0.03125–32	93.3	3.3	3.3
	Imipenem	16	16	8–16	0	0	100
	Ceftazidime	32	32	32– > 32	0	0	100
	Cefepime	32	32	16– > 32	0	0	100
	Amikacin	128	128	<1– > 128	33.9	0	66.1
	Ciprofloxacin	8	8	<0.0625– > 8	1.7	1.7	96.6
	Levofloxacin	16	16	<0.125– > 16	1.7	3.4	94.9
	Gentamicin	128	128	<1– > 128	18.6	0	81.4
*Citrobacter freundii*	Cefiderocol	0.03125	1	0.03125–2	100	0	0
	Imipenem	16	16	2–16	0	5.3	94.7
	Ceftazidime	64	64	8–64	0	0	100
	Ceftriaxone	64	64	32–64	0	0	100
	Cefepime	64	64	16–64	0	5.6	94.4
	Amikacin	16	64	2–64	36.8	0	63.2
	Ciprofloxacin	4	4	1–4	0	0	100
	Levofloxacin	8	8	0.5–8	5.3	10.5	84.2
	Tobramycin	16	16	8–16	0	0	100
	Gentamicin	16	16	1–16	12.5	6.3	81.3
*Klebsiella oxytoca*	Cefiderocol	0.03125		0.03125–1	100	0	0
	Imipenem	8		≤1–16	14.3	0	85.7
	Ceftazidime	64		≤1–64	14.3	0	85.7
	Ceftriaxone	64		≤1–64	20	0	80
	Cefepime	32		≤1–64	14.3	28.6	57.1
	Amikacin	2		≤2–64	42.9	14.3	42.9
	Ciprofloxacin	1		≤0.25–4	40	0	60
	Levofloxacin	1		≤0.25–8	28.6	14.3	57.1
	Tobramycin	8		≤1–16	40	0	60
	Gentamicin	16		≤1–16	40	0	60
*Pseudomonas aeruginosa*	Cefiderocol	1	16	0.03125–32	80	9.1	10.9
	Ceftazidime	4	64	1–64	59.3	14.8	25.9
	Ceftriaxone	64	64	32–64			
	Cefepime	8	64	1–64	68.5	13	18.5
	Amikacin	4	32	2–64	85.2	5.6	9.3
	Tobramycin	1	16	1–16	76.5	7.8	15.7
	Gentamicin	1	16	1–16	68.3	12.2	19.5
*Acinetobacter baumannii*	Cefiderocol	4	16	0.03125–16	73.3	16.3	10.5
	Imipenem	8	16	≤0.25–128	15.3	1.2	83.5
	Ceftazidime	64	64	4–64	6.1	0	93.9
	Ceftriaxone	2	64	0.5–64	56	2.7	41.3
	Cefepime	16	64	≤1–64	22.2	18.5	59.3
	Amikacin	4	64	≤0.25–64	61.6	0	38.4
	Ciprofloxacin	4	16	≤0.25–16	12.7	1.3	86.1
	Levofloxacin	8	16	≤0.25–64	10.8	0	89.2
	Tobramycin	16	64	≤1–64	12.8	2.6	84.6
	Gentamicin	64	64	≤1–64	7.7	5.1	87.2

MIC, minimum inhibitory concentration.

### 3.2 Antimicrobial efficacy of cefiderocol against carbapenem-resistant gram-negative bacterial isolates

Cefiderocol exhibits broad-spectrum antibacterial activity against CRGNB isolates. Specifically, *C. freundii* and *K. oxytoca* exhibited the highest susceptibility to cefiderocol (100.0%, resistance rates 0.0%), followed by *K. pneumoniae* (93.3% susceptible, 3.3% resistant), *E. hormaechei* (92.2% susceptible, 3.9% resistant), *E. coli* (76.8% susceptible, 7.1% resistant), and *P. aeruginosa* (80.0% susceptible, 10.9% resistant). *A. baumannii* demonstrated the lowest susceptibility, with 73.3% of isolates susceptible and 10.5% resistant. In stark contrast, other antibiotics, such as ceftazidime and ceftriaxone, exhibited high resistance levels, particularly with ceftazidime resistance in *E. coli* at 97.6% and ceftriaxone resistance in *E. hormaechei* at 100.0%.

**Figure 3:**
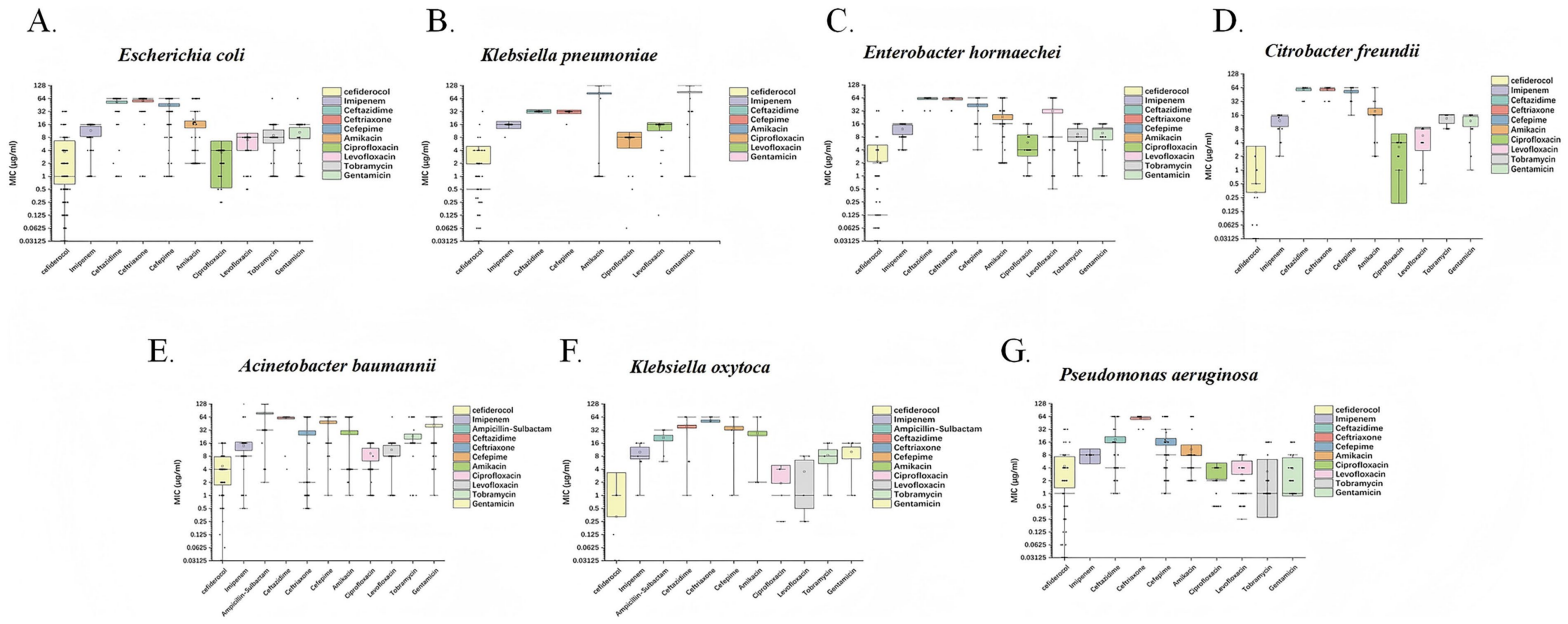
Box plots depicting the MIC (μg/mL) distributions of cefiderocol and other commonly used clinical antibiotics against seven CRGNB species. Each subplot corresponds to a distinct bacterial species: **(A)** *E. coli*, **(B)** *K. pneumoniae*, **(C)** *E. hormaechei*, **(D)** *C. freundii*, **(E)** *A. baumannii*, **(F)** *K. oxytoca*, and **(G)** *P. aeruginosa*. The x-axis represents different antibiotics, including cefiderocol, imipenem, ceftazidime, and others. The y-axis displays MIC values on a logarithmic scale, ranging from 0.03125 to 128 μg/mL.

Based on the MIC analysis of cefiderocol against various Gram-negative bacteria, the drug exhibited strong *in vitro* activity. For *P. aeruginosa*, cefiderocol shows effective inhibition, with a MIC_50_ of 1 μg/mL and MIC_90_ of 16 μg/mL. It is also effective against *A. baumannii*, with MIC_50_ at 4 μg/mL and MIC_90_ at 16 μg/mL, demonstrating good activity despite high resistance in this pathogen. *E. coli* showed high susceptibility with MIC_50_ of 1 μg/mL and MIC_90_ of 8 μg/mL. *E. hormaechei* and *K. pneumoniae* exhibited even higher sensitivity, with MIC_50_ values of 0.125 μg/mL and MIC_90_ of 0.5 μg/mL, respectively, highlighting excellent effectiveness. *C. freundii* and *K. oxytoca* were completely susceptible, with MIC_50_ values as low as 0.03125 μg/mL, emphasizing the potent activity of cefiderocol (Table 1; Figure 3).

### 3.3 Efflux pumps role in cefiderocol resistance using CCCP

Among the cefiderocol-resistant CRGNB strains, CCCP significantly reduced the MIC values of cefiderocol, with MICs for some strains dropping to below 0.03125 μg/mL, representing a reduction of over fourfold. In contrast, MIC values after inhibition with reserpine showed minimal changes (fold reduction near 1). However, in *P. aeruginosa* 4,087 and *A. baumannii* 10,521, the MIC values decreased by approximately twofold (Table 2).

**Table 2:** Cefiderocol minimum inhibitory concentration values of resistant isolates in the presence or absence of 50 μg/mL CCCP or reserpine.

Species	Isolates	MIC of cefiderocol (μg/mL)	MIC of cefiderocol in presence of CCCP MIC (μɡ/mƖ)	MIC of cefiderocol in presence of reserpine MIC (μɡ/mƖ)	MIC fold reduction of cefiderocol + CCCP	MIC fold reduction of cefiderocol +reserpine
	ATCC25922	<0.03125	<0.03125			
*Pseudomonas aeruginosa*	4,087	32	0.25	16	>4	2
	19,686	16	<0.03125	16	>4	1
	12,218	16	<0.03125	16	>4	1
	17,774	16	<0.03125	16	>4	1
	3,077	16	<0.03125	16	>4	1
	2,745	16	<0.03125	16	>4	1
*Acinetobacter baumannii*	10,521	32	<0.03125	16	>4	2
	11,253	16	<0.03125	16	>4	1
	12,076	16	<0.03125	16	>4	1
	12,150	32	<0.03125	32	>4	1
	12,439	16	<0.03125	16	>4	1
	14,118	32	<0.03125	32	>4	1
	14,179	16	<0.03125	16	>4	1
	14,184	32	<0.03125	16	>4	2
*Escherichia coli*	3,034	16	<0.03125	16	>4	1
	14,449	32	<0.03125	16	>4	2
	12,010	32	<0.03125	32	>4	1
	14,109	32	<0.03125	32	>4	1
	14,334	32	<0.03125	32	>4	1
	15,503	32	<0.03125	32	>4	1
	16,769	32	<0.03125	32	>4	1
*Enterobacter hormaechei*	1707	32	<0.03125	32	>4	1
	14,787	32	<0.03125	32	>4	1
*Klebsiella pneumoniae*	13	32	<0.03125	32	>4	1
	19	16	<0.03125	16	>4	1

### 3.4 Correlation between *β*-lactamase gene and cefiderocol resistance

**Figure 4:**
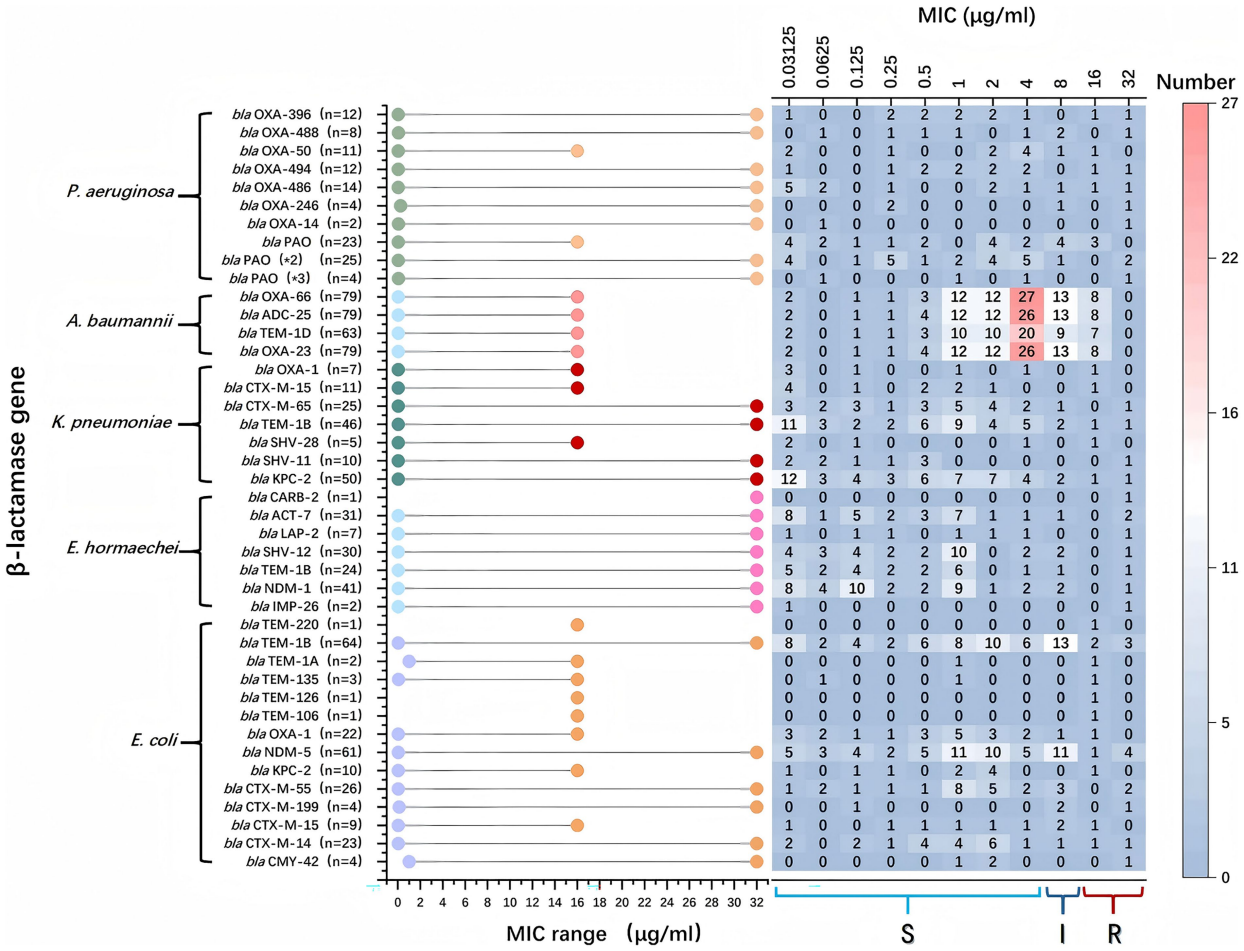
*β*-lactamase genes and their association with their minimum inhibitory concentration (MIC) ranges within various bacterial species. On the left, bacterial species and the specific *β*-lactamase genes they harbor are listed. The middle part of the chart uses colored dots to indicate the MIC ranges for bacteria with specific genes. The x-axis represents the MIC values in μg/ml, ranging from 0.03125 to 32. A higher MIC indicates stronger antibiotic resistance. The right section displays a heatmap, showing the distribution of bacterial strains at various MIC concentrations. Each cell contains the number of strains found at that MIC level, and the color gradient from light to dark indicates the strain count. Red represents the highest numbers, while blue represents lower numbers.

Among *P. aeruginosa* strains harboring *bla*_PAO_, 13.0% exhibited cefiderocol resistance. In contrast, resistance associated with other β-lactamase genes was confined to individual isolates. *A. baumannii* strains harboring *bla*_OXA-23_, *bla*_OXA-66_, *bla*_TEM-1D_, and *bla*_ADC-25_ displayed MICs ranging from 0.03125 to 16 μg/mL, with most clustering at 4 μg/mL. *K. pneumoniae* strains carrying *bla*_KPC-2_ displayed a broader MIC range (0.03125–32 μg/mL), but most were concentrated around 0.03125 μg/mL. *E. hormaechei* exhibited MICs between 0.03125 and 32 μg/mL, with only one resistant strain and the rest remaining susceptible. Similarly, *E. coli* strains carrying *bla*_TEM-1B_ and *bla*_NDM-1_ had MICs predominantly at 8 μg/mL, while those harboring other *β*-lactamase genes remained susceptible (Figure 4).

The predominant β-lactamase genes varied across species: In *P. aeruginosa*, *bla*_PAO_ and *bla*_OXA_ variants were most common; In *A. baumannii, bla*_OXA-23_, *bla*_OXA-66_, *bla*_TEM-1D_, and *bla*_ADC-25_ predominated. In *E. coli*, *bla*_NDM-5_, *bla*_TEM-1B_, and *bla*_CTX-M_ were the main genes, and in *K. pneumoniae*, *bla*_KPC-2_, *bla*_SHV_, *bla*_TEM-1B_, and various *bla*_CTX-M_ variants were prevalent (Table 3).

**Table 3:** Antimicrobial resistance profile and resistance genes of carbapenem-resistant gram-negative bacteria isolates resistant to cefiderocol.

Isolates	MIC (μg/ml)									Aminoglycoside resistance gene	Fluoroquinolone resistance gene	Carbapenemase gene	Other β-lactamase gene
	Cefiderocol	CAZ	CRO	FEP	AMK	CIP	LVX	TOB	GEN				
*Pseudomonas aeruginosa*													
4,087	32 (R)	64 (R)	64	64 (R)	4 (S)	4 (R)	8 (R)	16 (R)	16	*aph (3′)-Iib*, *aadA1*			*bla*_PAO_(*3), *bla*_OXA-14_, *bla*_OXA-488_
19,686	32 (R)	64 (R)		32 (R)	64 (R)	4 (R)	8 (R)	16 (R)		*ant (2″)-Ia*, *aadA24*, *aac (6′)-II*, *aac (6′)-IIa*, *aph (3′)-Iib*			*bla*_PAO_(*2), *bla*_OXA-246_, *bla*_OXA-396_, *bla*_OXA-494_
12,218	32 (R)	4 (S)			32 (I)	2 (R)	4 (R)	2 (I)	8	*aph (3′)-IIb*			*bla*_PAO_(*2), *bla*_OXA-486_
17,774	16 (R)	2 (S)		2 (S)	2 (S)		1 (S)	1 (S)		*aph (3′)-IIb*			*bla*_PAO_, *bla*_OXA-486_
3,077	16 (R)									*aph (3′)-IIb*			*bla*_PAO_, *bla*_OXA-494_, *bla*_OXA-396_
2,745	16 (R)	16 (I)	64	16 (I)	2 (S)	0.5 (S)	1 (S)	1 (S)	1	*aph (3′)-IIb*			*bla*_PAO_, *bla*_OXA-50_
*Acinetobacter baumannii*													
10,521	16 (R)	64 (R)	64 (R)	64 (R)	64 (R)	4	8 (R)	16 (R)	16 (R)	*armA*, *aph (3′)-Ia*		*bla*_OXA-23_, *bla*_OXA-66_	*bla*_TEM-1D_, *bla*_ADC-25_
11,253	16 (R)	64 (R)	64 (R)	64 (R)	4 (S)	4	8 (R)	1 (S)	4 (S)			*bla*_OXA-23_, *bla*_OXA-66_	*bla*_TEM-1D_, *bla*_ADC-25_
12,076	16 (R)	64 (R)	64 (R)	64 (R)	64 (R)	4	8 (R)			*armA*, *aph (6)-Id*		*bla*_OXA-23_, *bla*_OXA-66_	*bla*_ADC-25_
12,150	16 (R)	64 (R)	64 (R)	64 (R)	64 (R)	4	8 (R)	16 (R)	16 (R)	*armA*, *aph (6)-Id*, *aph (3′)-Ia*		*bla*_OXA-23_, *bla*_OXA-66_	*bla*_TEM-1D_, *bla*_ADC-25_
12,439	16 (R)	64 (R)	64 (R)	64 (R)	64 (R)	4	8 (R)	16 (R)	16 (R)	*armA*, *aph (6)-Id*, *aph (3′)-Ia*		*bla*_OXA-23_, *bla*_OXA-66_	*bla*_TEM-1D_, *bla*_ADC-25_
14,118	16 (R)		1 (S)		4 (S)	16			64 (R)	*armA*, *aph (6)-Id*, *aph (3′)-Ia*		*bla*_OXA-23_, *bla*_OXA-66_	*bla*_TEM-1D_, *bla*_ADC-25_
14,179	16 (R)		1 (S)	16 (I)	4 (S)	16	16 (R)		64 (R)	*aac (3)-I*, *armA*, *aph (6)-Id*, *aph (3′)-Ia*		*bla*_OXA-23_, *bla*_OXA-66_	*bla*_TEM-1D_, *bla*_ADC-25_
14,184	16 (R)		1 (S)		4 (S)	16	16 (R)		64 (R)	*armA*, *aph (6)-Id*		*bla*_OXA-23_, *bla*_OXA-66_	*bla*_TEM-1D_, *bla*_ADC-25_
*Escherichia coli*													
3,034	16 (R)	64 (R)	64 (R)	64 (R)	2 (S)	4 (R)	8 (R)		1 (S)	*rmtB*, *aac (6′)-Ib-cr*	*aac (6′)-Ib-cr*		*bla*_TEM-220_, *bla*_TEM-135_, *bla*_TEM-126_, *bla*_TEM-106_, *bla*_TEM-1B_, *bla*_CTX-M-14_
14,449	16 (R)	16 (R)	64 (R)	64 (R)	64 (R)	4 (R)	8 (R)	16 (R)	16 (R)	*rmtB*, *aac (6′)-Ib-cr*	*aac (6′)-Ib-cr*	*bla*_KPC-2_	*bla*_TEM-1A_, *bla*_CTX-M-15_, *bla*_OXA-1_
12,010	32 (R)	64 (R)	64 (R)	64 (R)	2 (S)	4 (R)	8 (R)	16 (R)	16 (R)	*aac (3)-IId*		*bla*_NDM-5_	*bla*_TEM-1B_, *bla*_CTX-M-14_
14,109	32 (R)	64 (R)	64 (R)	64 (R)	64 (R)	4 (R)	8 (R)	1 (S)	16 (R)	*rmtB*, *aac (6′)-Ib-cr*	*aac (6′)-Ib-cr*	*bla*_NDM-5_	*bla*_TEM-1B_, *bla*_CTX-M-199_
14,334	16 (R)	64 (R)	64 (R)	64 (R)	2 (S)	4 (R)	8 (R)	16 (R)	1 (S)	*rmtB*		*bla*_NDM-5_	*bla*_TEM-1B_
15,503	32 (R)	64 (R)	64 (R)	64 (R)	2 (S)	4 (R)	8 (R)		16 (R)	*aac (3)-IIa*		*bla*_NDM-5_	*bla*_TEM-1B_, *bla*_CTX-M-55_
16,769	32 (R)									*rmtB*		*bla*_NDM-5_	*bla*_CTX-M-55_, *bla*_CMY-42_
*Enterobacter hormaechei*													
1707	32 (R)									*aac (3)-IId*, *aadA2*, *aph (3′)-Ia*		*bla*_IMP-26_	*bla*_TEM-1B_, *bla*_LAP-2_, *bla*_CARB-2_, *bla*_ACT-7_
14,787	32 (R)				64 (R)	8 (R)	64 (R)			*aac (3)-IId*, *aac (6′)-Ib-cr*, *aadA16*	*aac (6′)-Ib-cr*	*bla*_NDM-1_	*bla*_SHV-12_, *bla*_ACT-7_
*Klebsiella pneumoniae*													
13	32 (R)			>32 (R)	>128 (R)	>8 (R)	>16 (R)		>128 (R)	*rmtB*		*bla*_KPC-2_	*bla*_SHV-11_, *bla*_TEM-1B_, *bla*_CTX-M-65_
19	16 (R)			>32 (R)	>128 (R)	>8 (R)	>16 (R)		>128 (R)	*aac (3)-Iid*, *rmtB*		*bla*_KPC-2_	*bla*_SHV-28_, *bla*_TEM-1B_, *bla*_CTX-M-15_, *bla*_OXA-1_

CAZ, ceftazidime; CRO, ceftriaxone; FEP, cefepime; AMK, amikacin; CIP, ciprofloxacin; LVX, levofloxacin; TOB, tobramycin; GEN, gentamicin; MIC, minimum inhibitory concentration. Copy number variants are denoted with asterisks (*).

The association between β-lactamase genes and cefiderocol resistance indicated a potential correlation between *bla*_OXA-66_ (*p =* 0.004) and *bla*_OXA-23_ (*p =* 0.050) in *A. bumannii*, and *bla*_SHV-12_ (*p =* 0.022) in *E. hormaechei* with cefiderocol resistance (Table 4). In contrast, other tested genes did not show a statistically significant correlation with resistance.

**Table 4:** Association between β-lactamase/carbapenemase genes and cefiderocol resistance analyzed by Mann–Whitney U rank-sum test.

Species	Form	Gene	*p*-value
*Escherichia coli*	Carbapenemase gene	*bla*_KPC-2_	0.175
		*bla*_NDM-1_	0.967
	β-lactamase gene	*bla*_TEM-220_	–
		*bla*_TEM-1A_	–
		*bla*_TEM-1B_	0.172
		*bla*_CTX-M-55_	0.361
		*bla*_TEM-135_	0.496
		*bla*_CTX-M-15_	0.282
		*bla*_CTX-M-14_	0.739
		*bla*_CTX-M-199_	0.065
		*bla*_CMY-42_	0.141
		*bla*_TEM-126_	–
		*bla*_OXA-1_	0.926
		*bla*_TEM-106_	–
*Klebsiella pneumoniae*	β-lactamase gene	*bla*_SHV-11_	0.925
		*bla*_SHV-28_	0.571
		*bla*_TEM-1B_	0.349
		*bla*_CTX-M-65_	0.161
		*bla*_CTX-M-15_	0.812
		*bla*_OXA-1_	0.699
	Carbapenemase gene	*bla*_KPC-2_	0.696
*Pseudomonas aeruginosa*	β-lactamase gene	*bla*_OXA-14_	0.345
		*bla*_OXA-246_	0.213
		*bla*_OXA-486_	0.918
		*bla*_OXA-494_	0.253
		*bla*_OXA-50_	0.194
		*bla*_OXA-488_	0.161
		*bla*_OXA-396_	0.415
*Acinetobacter baumannii*	Carbapenemase gene	*bla*_OXA-66_	0.004
	β-lactamase gene	*bla*_OXA-23_	0.050
		*bla*_TEM-1D_	0.534
		*bla*_ADC-2_	0.091
*Enterobacter hormaechei*	β-lactamase gene	*bla*_TEM-1B_	0.451
		*bla*_SHV-12_	0.022
		*bla*_LAP-2_	0.079
		*bla*_ACT-7_	0.415
		*bla*_CARB-2_	–
	Carbapenemase gene	*bla*_NDM-1_	0.576

### 3.5 Comparison of gene mutations in cefiderocol-resistant and cefiderocol-sensitive CRGNB

In *K. pneumoniae*, a missense mutation (c.442C > T, p.Pro148Ser) was detected in *envZ*, which encodes an osmoregulatory sensor protein (*p* = 0.036). In *E. coli*, multiple mutations were identified, particularly in *cirA*, encoding the iron-catecholate outer membrane transporter. Frameshift and missense mutations in *cirA* included c.1540_1545delC (p.Arg514fs, *p* = 0.006), c.1519_1535delG (p.Glu507fs, *p* = 0.006), and c.1395A > C (p.Glu465Asp., *p* = 0.004). Additionally, a frameshift mutation (c.268_269delAG, p.Ser90fs) was strongly associated with cefiderocol resistance (*p* < 0.001). Furthermore, a missense mutation in *nuoC* (c.1683_1685delC, p.Phe562Tyr) was identified (*p =* 0.006), suggesting a potential link between NADH-quinone oxidoreductase function and resistance. In *E. hormaechei*, mutations in *ampC* included c.953_956delTGGTinsCGGC (p.Val318Ala) and c.172C > T (p.Pro58Ser) (Table 5). These mutations were significantly more common in resistant strains (*p* < 0.05).

**Table 5:** Significant gene mutations in cefiderocol-resistant and -susceptible carbapenem-resistant gram-negative.

Species	Gene	Resistance mechanism	Variant type	Coding DNA mutation	Amino acid change	Mutation frequency in resistant strains	Mutation frequency in sensitive strains	*p*-value
*E. coli*	*cirA*	Iron-catecholate outer membrane transporter CirA	Frameshift variant & missense variant	c.1540_1545delCGCATAinsTGTG	p.Arg514fs	71.43%	18.06%	0.006
*E. coli*	*cirA*	Iron-catecholate outer membrane transporter CirA	Frameshift variant & missense variant	c.1519_1535delGAGACGGGCGCTAACGGinsACAGCGA	p.Glu507fs	71.43%	18.06%	0.006
*E. coli*	*cirA*	Iron-catecholate outer membrane transporter CirA	Missense variant	c.1395A > C	p.Glu465Asp	71.43%	16.67%	0.004
*E. coli*	*cirA*	Iron-catecholate outer membrane transporter CirA	Frameshift variant	c.268_269delAG	p.Ser90fs	71.43%	5.56%	0.000
*E. coli*	*nuoC*	NADH:quinone oxidoreductase subunit CD	Missense variant	c.1683_1685delCTTinsTTA	p.Phe562Tyr	28.57%	81.94%	0.006
*E. hormaechei*	*ampC*	Cephalosporin-hydrolyzing class C beta-lactamase ACT-61	Missense variant	c.953_956delTGGTinsCGGC	p.ValVal318AlaAla	50.00%	0.00%	0.044
*E. hormaechei*	*ampC*	Cephalosporin-hydrolyzing class C beta-lactamase ACT-62	Missense variant	c.172C > T	p.Pro58Ser	100.00%	9.30%	0.015
*K. pneumoniae*	*envZ*	Osmolarity sensor protein envZ	Missense variant	c.442C > T	p.Pro148Ser	50.00%	0.00%	0.036

### 3.6 Comparison of gene loss in cefiderocol-resistant and cefiderocol-susceptible CRGNB

In *P. aeruginosa*, the iron-uptake receptor gene *piuA* showed a significantly higher genomic absence frequency in cefiderocol-resistant isolates (66.7%) compared with susceptible strains (43.9%), suggesting that loss of this gene may contribute to cefiderocol resistance. Other findings included rare *ampD* losses in *P. aeruginosa*, retention of *ompK36* in *K. pneumoniae*, and minimal gene losses in *A. baumannii* and *E. coli*.

### 3.7 Differential expression of iron transporter-associated genes correlates with cefiderocol susceptibility across CRGNB

**Figure 5:**
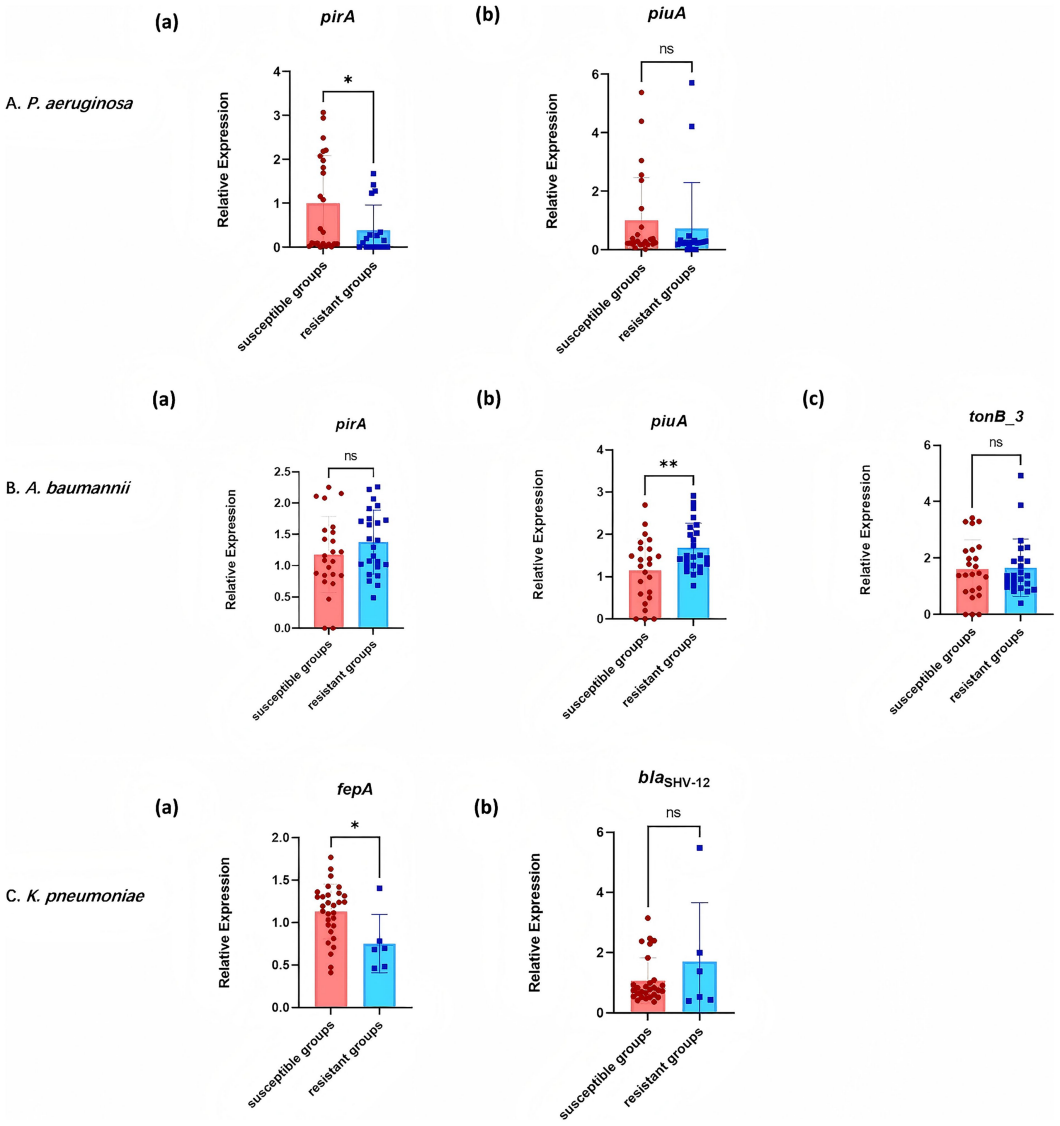
Expression of resistance genes in cefiderocol-resistant and cefiderocol-susceptible strains. **(A)** *P. aeruginosa*: Expression levels of *pirA* and *piuA* in resistant and susceptible groups. **(B)** *A. baumannii*: Expression levels of *pirA*, *piuA*, and *tonB_3* in different groups. **(C)** *K. pneumoniae*: Expression levels of *fepA* and *bla*_SHV-12_ in resistant and susceptible strains. * *p* < 0.05, ** *p* < 0.01, *** *p* < 0.001.

RT-qPCR analysis of resistance gene expression revealed differential expression across CRGNB strains (Figure 5). In *P. aeruginosa*, the siderophore synthase gene *pirA* exhibited marked downregulation in resistant isolates compared to susceptible counterparts [*p* < 0.05; Figure 5A (a)]. In contrast, the ferric iron receptor gene *piuA* did not show a statistically significant difference in expression between the two groups [*p >* 0.05; Figure 5A (b)].

In *A. baumannii*, an opposite trend was observed at the transcriptional level, with resistant strains exhibiting significant upregulation of *piuA* compared to susceptible strains [*p <* 0.01; Figure 5B (b)], while *pirA* expression remained statistically comparable across susceptibility groups [*p >* 0.05; Figure 5B (a)]. Moreover, the TonB-dependent energy transduction component *tonB_3* maintained stable expression regardless of resistance status [*p >* 0.05; Figure 5B (c)].

For *K. pneumoniae*, the expression of the enterobactin transporter gene *fepA* was significantly downregulated in resistant isolates [*p <* 0.05; Figure 5C (a)], whereas the *β*-lactamase gene *bla*_SHV-12_ exhibited conserved transcriptional activity between resistant and susceptible groups [*p >* 0.05; Figure 5C (b)].

### 3.8 Phylogenetic analysis of CRGNB

**Figure 6:**
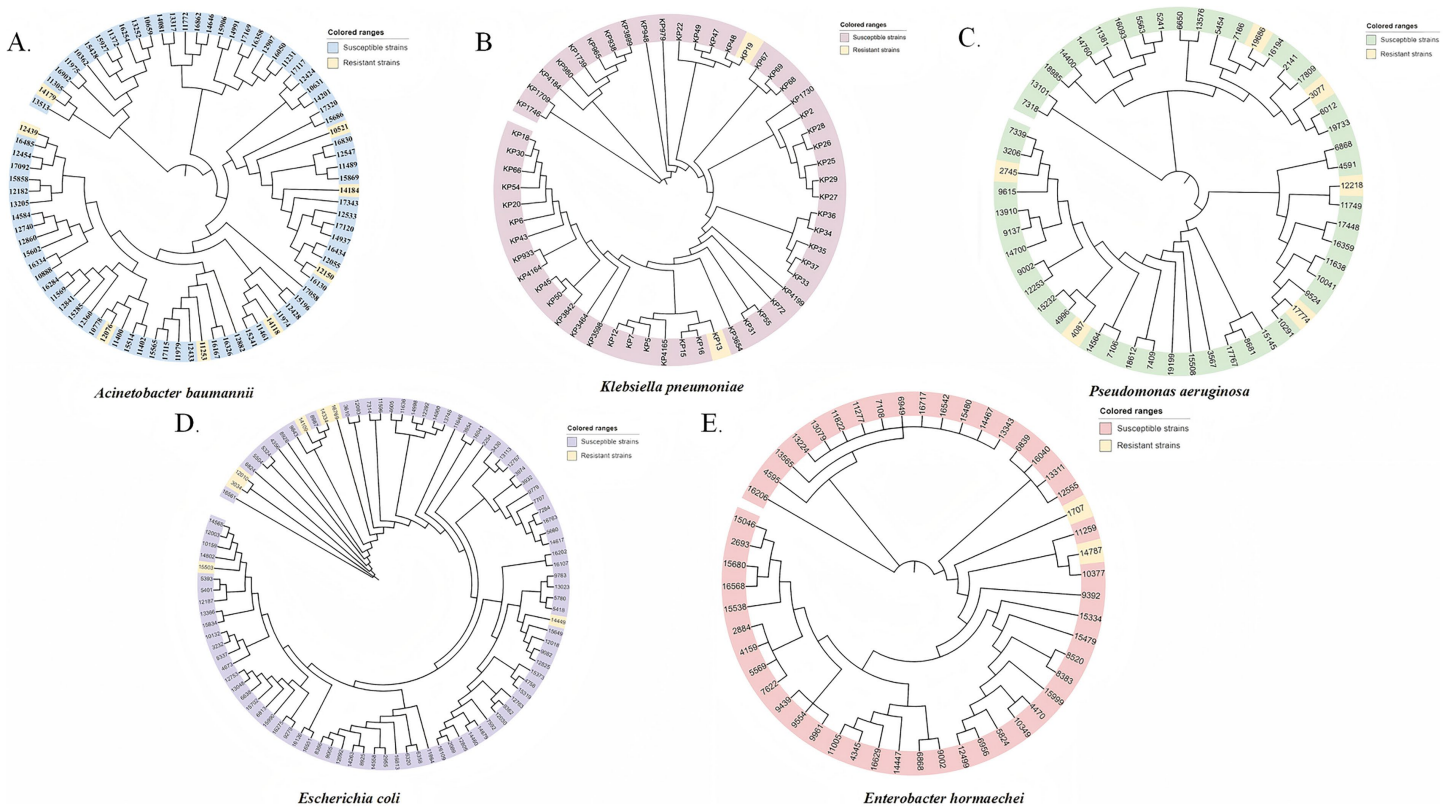
Phylogenetic trees of cefiderocol-resistant and susceptible CRGNB isolates based on SNP analysis. Phylogenetic trees were constructed for five major Gram-negative bacterial species, including **(A)** *A. baumannii*, **(B)** *K. pneumoniae*, **(C)** *P. aeruginosa*, **(D)** *E. coli*, and **(E)** *E. hormaechei*. Each isolate is represented at the tip of the tree and is color-coded based on its phenotypic susceptibility: resistant strains (yellow) and susceptible strains (gray or species-specific color tones). The clustering of resistant strains in certain branches indicates potential clonal dissemination. Scale bars represent the number of nucleotide substitutions per site.

Phylogenetic analysis indicated that the cefiderocol-resistant isolates of *K. pneumoniae*, *P. aeruginosa, A. baumannii*, and *E. hormaechei* did not cluster closely on the phylogenetic tree, indicating a lack of clonal relatedness among these strains analyzed (Figure 6). Thus, the same mutation within the specific genes may not be caused by clonal dissemination.

## 4 Discussion

The emergence of CRGNB represents a critical challenge to global public health, as these pathogens display extensive drug resistance and readily acquire new mechanisms. Our study provides localized evidence from China, highlighting both the potent *in vitro* activity of cefiderocol and the diversity of resistance determinants that may undermine its efficacy.

Cefiderocol demonstrated broad-spectrum antibacterial activity, inhibiting 83.0% of isolates at 4 μg/mL and achieving complete inhibition at 32 μg/mL. Consistent with previous study, cefiderocol inhibited 92.1% of CRE, 86.5% of CRKP isolates, and 88.9% of CRAB isolates at similar MIC levels (Mushtaq et al., 2020). Such a high efficacy, particularly at lower concentrations, is largely attributed to its unique mechanism of action—exploiting iron–siderophore uptake systems to traverse the bacterial outer membrane—which enables it to overcome resistance mechanisms that limit traditional antibiotics such as imipenem and ceftazidime (Kaye et al., 2023).

Furthermore, our results are consistent with international studies reporting high cefiderocol efficacy against CRE and *A. baumannii*. Despite this potent activity, we observed slightly higher MIC₉₀ values for *E. coli* and *K. pneumoniae* compared with data from the U. S. and Italy (Albano et al., 2020; Padovani et al., 2023), and our study further revealed variability in MIC values that likely reflects regional differences in resistance patterns and testing methodologies. For example, *K. pneumoniae* in our study showed MIC₅₀ and MIC₉₀ values of 0.5 μg/mL and 4 μg/mL, which were slightly lower than those reported in another study from China (MIC₅₀ = 2 μg/mL, MIC₉₀ = 4 μg/mL) (Zhao et al., 2023). At the species level, *C. freundii* and *K. pneumoniae* exhibited the highest susceptibility (100.0 and 93.3%, respectively), whereas *P. aeruginosa* and *A. baumannii* showed comparatively lower rates (80.0 and 73.3%). These findings suggest species-specific differences in susceptibility that may reflect distinct resistance mechanisms, including efflux pump activity and outer membrane permeability changes. Importantly, efflux pump inhibition assays confirmed that proton-driven pumps play a major role in cefiderocol resistance, as CCCP restored susceptibility in resistant isolates by actively expelling a broad range of antibiotics and lower intracellular drug concentrations (Ren et al., 2024), emphasizing the central role of efflux activity (Brepoels et al., 2022). Furthermore, the distinct effects observed between reserpine (targeting ATP-dependent pumps) and CCCP (disrupting proton-driven pumps) suggest that proton-driven efflux systems are particularly critical in mediating cefiderocol resistance (Kabra et al., 2019).

Genomic and transcriptional analyses provided further resolution into resistance mechanisms. *β*-lactamase genes were widely distributed: *bla*_PAO_ and various *bla*_OXA_ variants in *P. aeruginosa*; *bla*_OXA-23_, *bla*_OXA-66_, and *bla*_TEM-1D_ in *A. baumannii*; and *bla*_NDM-5_ and *bla*_CTX-M_ in *E. coli*. Notably, cefiderocol resistance in *E. hormaechei* was associated with *bla*_SHV-12_, echoing prior evidence that amplification of this gene can drive resistance in *K. pneumoniae* (Liu et al., 2024). Moreover, mutations in *ampC* (p.Val318Ala and p.Pro58Ser) and *envZ* (regulator of outer membrane porin expression through the OmpR/EnvZ system) were significantly enriched in resistant isolates, reinforcing the role of regulatory and enzymatic pathways in shaping cefiderocol susceptibility (Shields et al., 2020; Yang et al., 2024). These findings are consistent with studies that reported *ampC* mutations mediating cross-resistance to ceftazidime/avibactam and cefiderocol, as well as *envZ* alterations modulating porin expression (Dahdouh et al., 2024).

Iron transport pathways also emerged as critical modulators of resistance. In *P. aeruginosa*, genomic loss of *piuA* was more frequent in resistant isolates (66.7%) than in susceptible strains (43.9%), suggesting that gene deletion disrupts siderophore-mediated uptake of cefiderocol (Moynie et al., 2017). Conversely, in *A. baumannii*, *piuA* was transcriptionally upregulated in resistant strains, consistent with reports that aberrant expression of siderophore receptors can alter drug transport dynamics (Nishimura et al., 2022). In *K. pneumoniae*, downregulation of *fepA* was correlated with resistance, emphasizing the essential role of iron-uptake porins in facilitating cefiderocol entry (Daoud et al., 2022). Together, these data highlight the multifaceted interplay of *β*-lactamase activity, efflux systems, and iron transport alterations in cefiderocol resistance.

From a clinical perspective, our results reinforce the potential of cefiderocol as a critical therapeutic option for CRGNB infections in China. However, the variable activity across species and the strong association with efflux- and iron-related mechanisms suggest that susceptibility testing should be integrated into routine clinical practice before cefiderocol use. Furthermore, the ability of efflux pump inhibitors to restore susceptibility raises the possibility of novel combination strategies, though translation into clinical application will require careful evaluation of toxicity and pharmacokinetic constraints. Molecular surveillance incorporating *β*-lactamase variants, efflux activity, and iron-uptake pathways may improve the precision of empirical therapy, enabling pathogen-specific treatment strategies and helping to preserve cefiderocol’s clinical utility.

This study has several limitations. First, as it was conducted at a single tertiary hospital, the findings may not be generalizable to other regions with different resistance profiles. Second, some isolates were collected in earlier years, which may not fully capture the most recent epidemiological trends. Third, the cross-sectional design provides only a snapshot of susceptibility, and longitudinal surveillance will be needed to monitor evolving resistance patterns over time. Finally, we did not examine the expression of porins and penicillin-binding proteins (PBPs), which have been reported in other studies to contribute to cefiderocol resistance. Future studies incorporating multiple centers, prospective longitudinal sampling, and additional resistance mechanisms will be valuable to provide a more comprehensive understanding of cefiderocol resistance.

## 5 Conclusion

In summary, our findings confirm that cefiderocol is highly active against diverse CRGNB isolates in China, but resistance driven by β-lactamases, efflux systems, and iron-uptake alterations poses a serious challenge. Continuous surveillance, incorporation of molecular resistance markers into routine diagnostics, and consideration of species-specific responses will be essential to optimize the clinical use of cefiderocol.

## Data Availability

The datasets presented in this study can be found in online repositories. The names of the repository/repositories and accession number(s) can be found in the article/Supplementary material. All the sequences were deposited into the NCBI Sequence Read Archive (SRA). The Whole Genome Shotgun BioProject for these isolates has been deposited at GenBank, the BioProject number was PRJNA882512.

## Footnotes

^1^ http://www.genomicepidemiology.org
