# Supplementary material for: In vitro antimicrobial activity and resistance mechanisms of cefiderocol against clinical carbapenem-resistant gram-negative bacteria
Source: Front Microbiol. 2025 Oct 3;16:1670179. doi: 10.3389/fmicb.2025.1670179 (PMC12532133; doi:10.3389/fmicb.2025.1670179)
Supplement: Supplementary file 1 [file Table_1.docx]

**Supplementary Material**

Table S1. The Detailed Distribution of Isolation Sources for Each Bacterial Species

|  | *Escherichia coli*  (n=99) | *Klebsiella oxytoca*  (n=7) | *Acinetobacter baumannii*  (n=82) | *Citrobacter freundii*  (n=20) | *Enterobacter hormaechei*  (n=51) | *Pseudomonas aeruginosa*  (n=53) | *Klebsiella pneumoniae*  (n=58) |
| --- | --- | --- | --- | --- | --- | --- | --- |
| bile | 12 | 0 | 0 | 0 | 0 | 0 | 0 |
| urine | 39 | 0 | 0 | 8 | 6 | 0 | 10 |
| secretion | 10 | 0 | 0 | 1 | 9 | 0 | 1 |
| abdominal dropsy | 8 | 2 | 0 | 3 | 8 | 0 | 0 |
| sputum | 17 | 5 | 0 | 5 | 15 | 0 | 33 |
| blood | 11 | 0 | 82 | 1 | 8 | 53 | 2 |
| catheter | 1 | 0 | 0 | 0 | 3 | 0 | 0 |
| rectal swab | 1 | 0 | 0 | 0 | 0 | 0 | 0 |
| cerebrospinal fluid | 0 | 0 | 0 | 2 | 0 | 0 | 0 |
| pleural effusion | 0 | 0 | 0 | 0 | 2 | 0 | 1 |
| blank | 0 | 0 | 0 | 0 | 0 | 0 | 8 |
| Pus | 0 | 0 | 0 | 0 | 0 | 0 | 2 |
| Incision secretion | 0 | 0 | 0 | 0 | 0 | 0 | 1 |
