# Supplementary material for: In vitro antimicrobial activity and resistance mechanisms of cefiderocol against clinical carbapenem-resistant gram-negative bacteria
Source: Front Microbiol. 2025 Oct 3;16:1670179. doi: 10.3389/fmicb.2025.1670179 (PMC12532133; doi:10.3389/fmicb.2025.1670179)
Supplement: Supplementary file 2 [file Table_2.docx]

Table S2. GenBank Accession Numbers of Whole-Genome Sequences of Carbapenem-Resistant Gram-Negative Bacteria

| Accession Number | | | | |
| --- | --- | --- | --- | --- |
| JAVILM000000000 | JAVINW000000000 | JAVIKW000000000 | JAVILD000000000 | JANWQS000000000 |
| JAVIOF000000000 | JAVINJ000000000 | JAVILX000000000 | JANWRS000000000 | JANWSA000000000 |
| JAVILV000000000 | JAVIKZ000000000 | JAVILP000000000 | JANWOV000000000 | JANWOY000000000 |
| JAVIMR000000000 | JAVINZ000000000 | JAVILW000000000 | JANWPJ000000000 | JANWRP000000000 |
| JAVIMD000000000 | JAVIOB000000000 | JAVILQ000000000 | JANWPO000000000 | JANWPF000000000 |
| JAVIKS000000000 | JAVIOK000000000 | JAVILF000000000 | JANWRA000000000 | JANWOX000000000 |
| JAVIMW000000000 | JAVIME000000000 | JAVIKX000000000 | JANWOU000000000 | JANWPE000000000 |
| JAVIMV000000000 | JAVILT000000000 | JAVILR000000000 | JANWPK000000000 | JANWQN000000000 |
| JAVIKL000000000 | JAVINQ000000000 | JAVIMX000000000 | JANWRC000000000 | JANWQY000000000 |
| JAVINN000000000 | JAVIKT000000000 | JAVILS000000000 | JANWOM000000000 | JANWPY000000000 |
| JANWLV000000000 | JAVIKN000000000 | JAVILU000000000 | JANWRQ000000000 | JANWRH000000000 |
| JAVINL000000000 | JAVIMS000000000 | JAVILC000000000 | JANWPH000000000 | JANWPT000000000 |
| JAVIOG000000000 | JAVIOJ000000000 | JAVINK000000000 | JANWPQ000000000 | JANWRM000000000 |
| JAVILG000000000 | JAVIMB000000000 | JAVINR000000000 | JANWOR000000000 | JANWQE000000000 |
| JAVIMG000000000 | JAVIML000000000 | JAVILJ000000000 | JANWPP000000000 | JANWQC000000000 |
| JAVINB000000000 | JAVIOH000000000 | JAVILL000000000 | JANWQI000000000 | JANWPX000000000 |
| JAVILY000000000 | JAVIMA000000000 | JAVIOL000000000 | JANWQG000000000 | JANWQM000000000 |
| JAVIKR000000000 | JAVILZ000000000 | JAVINM000000000 | JANWQV000000000 | JANWQK000000000 |
| JAVILA000000000 | JANWLW000000000 | JAVIMU000000000 | JANWOW000000000 | JANWRL000000000 |
| JAVIMN000000000 | JAVIMM000000000 | JAVINE000000000 | JANWRT000000000 | JANWPS000000000 |
| JAVINU000000000 | JAVILH000000000 | JAVIMI000000000 | JANWRD000000000 | JANWRU000000000 |
| JAVIMJ000000000 | JAVIOD000000000 | JAVIND000000000 | JANWQP000000000 | JANWPZ000000000 |
| JAVINS000000000 | JAVIKO000000000 | JAVIKP000000000 | JANWPD000000000 | JANWRX000000000 |
| JAVING000000000 | JANWLT000000000 | JAVINP000000000 | JANWQX000000000 | JANWRF000000000 |
| JAVINV000000000 | JAVIOE000000000 | JAVILN000000000 | JANWRB000000000 | JANWPL000000000 |
| JAVINA000000000 | JAVIKQ000000000 | JAVIKM000000000 | JARBGW000000000 | JANWRI000000000 |
| JAVIMQ000000000 | JAVIMK000000000 | JANWMJ000000000 | JANWPR000000000 | JANWRW000000000 |
| JAVIOC000000000 | JAVIKV000000000 | JAVIOI000000000 | JANWQH000000000 | JANWQW000000000 |

| Accession Number (continued) | | | | |
| --- | --- | --- | --- | --- |
| JANWPG000000000 | JANWON000000000 | RZKQ00000000 | RZJY00000000 | JBBXIU000000000 |
| JANWOK000000000 | JANWOL000000000 | RZKP00000000 | RZLK00000000 | JBBXII000000000 |
| JANWQU000000000 | JANWOZ000000000 | RZLS00000000 | RZME00000000 | JBBXIP000000000 |
| JANWPN000000000 | JANWPA000000000 | RZLO00000000 | VEOP00000000 | JBBXJG000000000 |
| JANWRY000000000 | JACKXB000000000 | JANWMK000000000 | RZMH00000000 | JBBXIL000000000 |
| JANWQD000000000 | JANWQF000000000 | RZLE00000000 | RZMR00000000 | JBBXIW000000000 |
| JANWRO000000000 | JANWQL000000000 | RZKN00000000 | RZLD00000000 | JBBXIQ000000000 |
| JANWOJ000000000 | JANWOT000000000 | RZKS00000000 | RZLV00000000 | JBBXIO000000000 |
| JANWPV000000000 | JANWPC000000000 | RZKT00000000 | RZKH00000000 | JBDHLN000000000 |
| JANWRV000000000 | JANWPB000000000 | RZLM00000000 | RZMQ00000000 | JBBXIE000000000 |
| JANWPM000000000 | JANWRR000000000 | VEOL00000000 | RZKC00000000 | JBDHLJ000000000 |
| JANWRN000000000 | JANWRZ000000000 | RZKK00000000 | RZKL00000000 | JBBXIJ000000000 |
| JANWRG000000000 | JANWOP000000000 | RZKJ00000000 | VEON00000000 | JBBXIC000000000 |
| JANWQQ000000000 | RZMC00000000 | RZLA00000000 | RZML00000000 | JBBXIG000000000 |
| JANWPI000000000 | RZLC00000000 | RZKE00000000 | RZMJ00000000 | JBBXJL000000000 |
| JANWPW000000000 | RZKM00000000 | RZLL00000000 | RZMF00000000 | JBBXIR000000000 |
| JANWQZ000000000 | RZKY00000000 | JANWMH000000000 | RZKR00000000 | JBBXHX000000000 |
| JANWQJ000000000 | RZKX00000000 | RZLW00000000 | RZKU00000000 | JBDHLK000000000 |
| JANWQO000000000 | RZLX00000000 | RZKZ00000000 | JBBXJH000000000 | JBBXIZ000000000 |
| JARBGY000000000 | RZLU00000000 | RZMD00000000 | JBBXIF000000000 | JBDHLL000000000 |
| JANWRJ000000000 | RZJX00000000 | RZKB00000000 | JBBXHZ000000000 | JBDHLM000000000 |
| JANWQA000000000 | RZMO00000000 | VEOO00000000 | JBBXIA000000000 | JBBXHV000000000 |
| JANWPU000000000 | RZLB00000000 | RZKD00000000 | JBBXIH000000000 | JBBXJA000000000 |
| JARBGX000000000 | RZJW00000000 | RZKO00000000 | JBBXHS000000000 | JBBXJE000000000 |
| JANWQB000000000 | RZLN00000000 | RZLT00000000 | JBBXHR000000000 | JBBXJC000000000 |
| JANWOQ000000000 | RZJZ00000000 | RZKF00000000 | JBBXID000000000 | JBBXIS000000000 |
| JANWOS000000000 | RZKA00000000 | JANWMG000000000 | JBBXIM000000000 | JBBXHY000000000 |
| JANWQT000000000 | RZMK00000000 | RZLZ00000000 | JBBXJK000000000 | JBBXIV000000000 |

| Accession Number (continued) | | | |
| --- | --- | --- | --- |
| JBBXIX000000000 | JANWNJ000000000 | JANWNX000000000 | VEOQ00000000 |
| JBBXJI000000000 | JANWMO000000000 | JANWNK000000000 | JABEWO000000000 |
| JBBXIY000000000 | JANWNZ000000000 | JANWOA000000000 | RZMG00000000 |
| JBBXJB000000000 | JANWMT000000000 | JARBIC000000000 | RZMB00000000 |
| JBBXIK000000000 | JARBIB000000000 | JAVIMH000000000 | VEOR00000000 |
| JBBXHW000000000 | JANWNH000000000 | JAVINF000000000 | RZLH00000000 |
| JBBXIB000000000 | JANWOF000000000 | JAVIMF000000000 |  |
| JBBXHT000000000 | JANWML000000000 | JAVIOA000000000 |  |
| JBBXHU000000000 | JANWOG000000000 | JAVIMY000000000 |  |
| JBBXJJ000000000 | JANWNP000000000 | JAVILK000000000 |  |
| JBBXJD000000000 | JANWNY000000000 | JAVIKY000000000 |  |
| JBBXIN000000000 | JANWMY000000000 | JANWLU000000000 |  |
| JBBXIT000000000 | JANWMQ000000000 | JAVINO000000000 |  |
| JBBXJF000000000 | JANWMS000000000 | JAVILE000000000 |  |
| JANWOC000000000 | JANWNG000000000 | JAVIMO000000000 |  |
| JANWMN000000000 | JANWNI000000000 | JAVILO000000000 |  |
| JANWNV000000000 | JANWNO000000000 | JAVIMZ000000000 |  |
| JARBGZ000000000 | JANWNW000000000 | JAVINC000000000 |  |
| JANWNC000000000 | JANWNS000000000 | JABEWM000000000 |  |
| JANWMU000000000 | JANWOE000000000 | RZMI00000000 |  |
| JANWND000000000 | JANWNN000000000 | JABEWL000000000 |  |
| JANWNF000000000 | JANWNU000000000 | JABEWN000000000 |  |
| JANWMW000000000 | JANWMR000000000 | JABEWE000000000 |  |
| JANWMX000000000 | JANWOH000000000 | JABEWI000000000 |  |
| JANWOB000000000 | JANWNT000000000 | JANWOO000000000 |  |
| JANWMP000000000 | JANWMZ000000000 | RZKI00000000 |  |
| JANWNL000000000 | JANWMV000000000 | RZKG00000000 |  |
| JANWNE000000000 | JANWNQ000000000 | JABEWR000000000 |  |
