# Supplementary material for: In vitro antimicrobial activity and resistance mechanisms of cefiderocol against clinical carbapenem-resistant gram-negative bacteria
Source: Front Microbiol. 2025 Oct 3;16:1670179. doi: 10.3389/fmicb.2025.1670179 (PMC12532133; doi:10.3389/fmicb.2025.1670179)
Supplement: Supplementary file 3 [file Table_3.docx]

Table S3 Specific Primer Sequences Used in RT‒qPCR

|  | Primer name | Sequence(5’–3’) | Size of product(bp) | References |
| --- | --- | --- | --- | --- |
| *Pseudomonas aeruginosa* | *rpoD*-F | GGGCGAAGAAGGAAATGGTC | 21 | ^[1]^ |
|  | *rpoD*-R | CAGGTGGCGTAGGTAGAGAA | 20 | ^[1]^ |
|  | *PirA*-F | TACTTCAAGCGCGAGAACAACA | 22 | ^[2]^ |
|  | *PirA*-R | CCAGTTCGAGGTTACGGTTACG | 22 | ^[2]^ |
|  | *PiuA*-F | AACAAGACCGATTCGGACGAT | 21 | ^[2]^ |
|  | *PiuA*-R | GTGCCGTTGTTGTTCTGGGTA | 21 | ^[2]^ |
| *Acinetobacter baumannii* | *rpoB*-F | ACGCCTAAAGGTGAAACTCAGTTAA | 25 | ^[3]^ |
|  | *rpoB*-R | GTACCAGATGGAACACGTAAAGATG | 25 | ^[3]^ |
|  | *PirA*-F | GTCTATGGCTTTTGCTGCACA | 21 | ^[4]^ |
|  | *PirA*-R | GCGATTGCTTCACTTGCTCT | 20 | ^[4]^ |
|  | *PiuA*-F | CAGTTGGTGGCAGCATCAAT | 20 | ^[3]^ |
|  | *PiuA*-R | TGCTGCAATGCCATTTCCAA | 20 | ^[3]^ |
|  | *tonB3*-F | GCCAACGACTACTGTGAC | 18 | ^[5]^ |
|  | *tonB3*-R | GCTCCACTGTACACCTGA | 18 | ^[5]^ |
| *Klebsiella pneumoniae* | *rpoB*-F | CTTGGTACGACCGTTCACGT | 20 | ^[6]^ |
|  | *rpoB*-R | GCTGAAACTGAACCACCTGG | 20 | ^[6]^ |
|  | *bla*_SHV12_-F | AGCCGCTTGAGCAAATTAAA | 20 | ^[6]^ |
|  | *bla*_SHV12_-R | GCTGGCCAGATCCATTTCTA | 20 | ^[6]^ |
|  | *fepA*-F | CGACTTAAAAGCCGAGACCAGC | 22 | ^[7]^ |
|  | *fepA*-R | ATCTTGTTGCGGTAGTCGTTGC | 22 | ^[7]^ |

**References**

[1] BADAL D, JAYARANI A V, KOLLARAN M A, PRAKASH D, P M, SINGH V. Foraging Signals Promote Swarming in Starving Pseudomonas aeruginosa. mBio, 2021; 12 (5): e0203321.

[2] LUSCHER A, GASSER V, BUMANN D, MISLIN G L A, SCHALK I J, KOHLER T. Plant-Derived Catechols Are Substrates of TonB-Dependent Transporters and Sensitize Pseudomonas aeruginosa to Siderophore-Drug Conjugates. mBio, 2022; 13 (4): e0149822.

[3] FINDLAY J, BIANCO G, BOATTINI M, NORDMANN P. In vivo development of cefiderocol resistance in carbapenem-resistant Acinetobacter baumannii associated with the downregulation of a TonB-dependent siderophore receptor, PiuA. J Antimicrob Chemother, 2024; 79 (4): 928-930.

[4] ESCALANTE J, NISHIMURA B, TUTTOBENE M R, SUBILS T, MEZCORD V, ACTIS L A*, et al.* The Iron Content of Human Serum Albumin Modulates the Susceptibility of Acinetobacter baumannii to Cefiderocol. Biomedicines, 2023; 11 (2).

[5] ZIMBLER D L, ARIVETT B A, BECKETT A C, MENKE S M, ACTIS L A. Functional features of TonB energy transduction systems of Acinetobacter baumannii. Infect Immun, 2013; 81 (9): 3382-3394.

[6] LIU C, YI J, LU M, YANG P, DU C, JIANG F*, et al.* Dynamic within-host cefiderocol heteroresistance caused by bla(SHV-12) amplification in pandrug-resistant and hypervirulent Klebsiella pneumoniae sequence type 11. Drug Resist Updat, 2024; 73 101038.

[7] BRYAN E J, QIAO Q, WANG Y, ROBERGE J Y, LAVOIE E J, PILCH D S. A FtsZ Inhibitor That Can Utilize Siderophore-Ferric Iron Uptake Transporter Systems for Activity against Gram-Negative Bacterial Pathogens. Antibiotics (Basel), 2024; 13 (3).
